# Cases of Cerebro-Spinal Menengitis

**Published:** 1867-01

**Authors:** W. C. Moore

**Affiliations:** Atlanta, Ga.


### ARTICLE II.

*Cases of Cerebro-Spinal Menengitis.* W. C. Moore, M. D., Atlanta, Ga.

Case 1.—A young man aged 20, (attached to cavalry service,) was admitted in Fair Ground Hospital, No. 2, at this place, the latter part of May, 1864; was taken sick 2d day before, at New Hope church, about 11 A. M.; was examined by his surgeon, and sent in ambulance to Cartersville, and from thence to this place: before arriving at Cartersville, became delirious, in which condition he was received in the hospital, with the additional symptoms—face flushed, pupil of left eye dilated, right contracted, complete oposthotonos, hyperasthesia extreme, (two nurses were detailed to keep him in his bed,) pulse 100 and almost imperceptible, temperature of surface normal, bowels constipated, diathesis nervous. He was put on the following treatment: Blister, extending from one ear to the other in width at upper end; in length, from the hair to the last cervical vertebra. Prior to being applied, the surface was irritated with sinapism, and ol terebinth. Vesication occurred in about 2½ hours. In addition to this, sinapism was applied from lower edge of blister to the sacrum, extending two inches on either side of the spine, to remain on as long as possible without blistering. Ol terebinth. was then applied. The following was given internally: ℞ hyd. chlor. mit. gr. xv.; sul. quinia, pulv. doveri ā ā gr. xii.; M. & ft. chart. No. iii. s. One every hour, with ℥ ss. camph. tinc. opii. at bed time.

Saw him at 8 A. M., next day: was still restless, and much in the same condition as yesterday. No action from bowels. Voided urine involuntarily. Blister dressed with following: Equal parts of ungt. hydrar. and cerate simplex; to remain one hour, and cerate resin applied. Reapplied sinapism to the spine, to be followed by application of ol terebinth. every two hours. Ordered sul. mag. ℥ i., to be followed one hour after by enemata. In ten minutes after its administration copious dark watery operations took place, and were repeated every 30 minutes until 3 P. M. I saw they were exhausting him rapidly, and ordered pulv. opii. pulv. camphor, splumbi act. ā ā gr. ss. to be given every ½ hour. After taking two, the discharges became less frequent. The powders were stopped, with instructions to be again administered in case symptoms required. Ordered sherry wine with equal quantity of sweet milk every two hours.

Towards night patient became more quiet. Ordered ℥ ss. camphor. tinc. opii. at bed-time. Milk and wine to be kept up through the night.

Called 8 A. M. next day. Found him much better. Rested about 4 hours between midnight and day. Pulse 90, and more full. Muscles of neck and back considerably relaxed. Bowels moved twice during night. ℞ Pulv. dov., sul. quin. āā gr. xii., pulv. capsici gr. vi., m. et. ft. chart No. iii.—S. One every hour. Continue wine and milk freely. Continue resin cerate to blister and ol. terebinth. to spine. Towards evening he began to talk rationally. Asked for something to eat. Gave him arrow root boiled in milk, which has been his diet since admission. Ordered ℥ ss. camphor. tinc. opii. at bed-time, to be repeated in case he did not rest.

Called at 8 A. M. next day. Found him considerably improved. Pulse same as yesterday, both as to volume and frequency. No action from bowels. Was quiet all night, but did not sleep much until nearly day. Conversation rational. Strict orders to talk as little as possible to him, and keep ward perfectly quiet. Continue resin cerate to blister and ol. terebinth. to spine. Ordered ℞ hydrar. chlor. mit. quin. sul. āā gr. xiii., pulv. capici gr. vi., m. et. ft. chart No. iii.—S. One every hour. At 1 P. M. gave him ℥ ss. sul. mag. which operated without enema. Camphor tinc. opii ℥ ss. at bed-time. Continue diet, with wine and milk.

Called at 8 A. M. next day. Found patient considerably better. Pulse 80, and full. Said he was hungry. Allowed him two buttered batter-cakes, with a soft toast and cup of tea at each meal; in addition, a little chicken for breakfast, and soup for dinner. Dressed blister with cerate simplex. Spine with mixture of equal parts of ol. terebinth. and ol. olivia. Two actions on bowels last night. Continue milk and wine, with ℥ ss. paregoric at bed-time.

Called at 8 A. M. next day. Found him sitting up in bed. One action from bowels last night. Continue treatment and diet.

Called at 8 A. M. next day. Patient up and clothes on.

Allowed him to walk about the ward, but not sufficient to fatigue himself. Blister nearly healed. Continue diet and treatment.

Called at 8 A. M. next day. Found him up and walking about. Rests well at night. Appetite good. Consider him out of danger. Continue diet with the wine and milk. From this time on his diet was prudently increased, until his return to his command. ℥ ss. of the following mixture was given him at each meal, as a tonic: ℞ tinc. pinckney bark ℥ ii., tinc. ferri. chlor. ʒ iss., tinc. zingberis ℥ i.; m.

Case 2.—Was treated at Ocmulgee Hospital, Macon, Ga., in Sept. 1864. He was under charge of a med. officer, who informed me that he was received in hospital 3 days before, with congestive chill. (Malignant intermittent and remittent fever was then prevailing at Macon among those not acclimated.) The subject was a stout mulatto man, aged 30: apparently of a sanguine temperament. He died 3 days after with all the symptoms of Cerebro-Spinal Meningitis. Autopsy, 2 hours after death: Arachnoid congested, with effusion of plastic lymph over middle lobe left hemisphere of the cerebrum, causing adhesions. Pia mater greatly congested, especially between the convolutions: half pint of serum was discharged from ventricles and folds of arachnoid. Dura water congested at and near lateral and cavernous sinuses. Substance of brain apparently healthy.

Case 3.—Admitted and treated at same hospital as No. 2. Subject a stout black man, aged about 25, of a strumous Diathesis. Six days prior to death was admitted in hospital with double pneumonia. He continued to grow worse for three days, when he commenced to complain of severe pain of the head, and in twelve hours was delirious, with great hyperasthesia, in which condition he died.

Autopsy: Four hours after death " all the membranes of the brain congested, with effusion of serum in ventricles, and between folds of arachnoid. Great congestion at lateral sinuses and base of brain. Both lungs in a state of red hepatization, except upper lobes. Pleuritic adhesions on both sides, with effusion in pleural cavity."

Case 4.—This case occurred in this city, in March, 1866. The subject was a negro girl, aged 8, of strumous diathesis. Was first attacked with influenza: in three or four days became delirious. An intelligent physician was requested to see her, and made two or three visits and quit. About ten days after its being attacked with influenza I was called to see it, and found it in the following condition: Decubitas dorsal; emaciation great; would not answer when spoken to; complete rigidity of cervical and dorsal muscles; pupils of both eyes dilated; eyes injected; bowels constipated; pulse one hundred, and very weak; temperature of skin normal.

Prescribed blister to back of neck, to be followed by application of resin cerate: sinapism whole length of spine, to be followed with oil terebinth. ℞ Hydrar. chlor. mit. quin. sul. ā a gr. xii., pulv. doveri. gr. vi. m. ft. chart iii. S. One every hour; to be followed four hours after last dose, with ℥ ss. ol. ricini, which produced three or four dark operations by midnight.

Called next day. Patient much the same. Ordered ℞ quin. valeriante gr. viii., ext. hyoscyamus gr. iv., bismuth sub. nit. gr. xii., tinc. aconiti rad. gtt. x. m. ft. pil. No. iv. S. One every two hours. Continue dressing to blister and spine. ℥ ii. paregoric, with hot pediluvia at bed-time. Diet milk and rice.

Call next morning. Patient not so restless last night, but did not sleep much. One action from bowels last night. Continue treatment, with addition of sherry wine and milk.

Call next morning. Patient had two actions from bowels last night. Micturation free. Pulse 95, and more full. Delirium continues. Pupils still dilated. Continue treatment.

Call next morning. Patient about same as yesterday. Treatment and diet continued.

The next morning the patient was a little better: could talk rational: wanted to get up. Strict orders enjoined not to take her up, nor talk much to her; but keep the room quiet. Continue treament and diet.

Called next day. Found her much worse. (Her mother, contrary to orders, had allowed her to get up and sit by the fire an hour.) Had one spasm last night. Ordered sinapism to the spine; to be followed with applications of oil terebinth. ℞ Hydrar. chlor. mit. gr. xii., quin. sul. gr. vi., ext. hyoscyamus gr. iii., m. ft. pil. iii. S. One every hour; to be followed in two hours after last, with oil ricini ℥ ii. This produced three or four evacuations by bed-time. Camp. tinc. opii. ʒ ii. at bed-time, with hot pediluvia.

Call next morning. Found her in a low mnttering, delirious condition. Renewed pills of quin. valer. hyoscyamus aconite and bismuth, with wine and milk.

Call second day after. Found her alive, much to my surprise. Continue treatment and diet.

Call next day. Her mother having to seek a new home, was getting ready to remove this patient to the freedmen's hospital. This terminated my connection with the case. I learned from the medical officer that she was in a comatose condition when admitted, and died the sixth day thereafter. The following developments were disclosed by a post mortem examination: "All the membranes congested, but no effusion of plastic lymph. Found pus and serum in ventricles. Substance of brain softened, with partial disorganization about the base.

Etiology.—As to the causes of this disease, I profess to know but little. Various opinions have been expressed, attributing it to cold, malaria, and other causes. All of which I consider mere theory, until I see more positive evidence in relation thereto. Its pathology we find from dissection. I consider it a disease *sui generis*, attacking the cerebro-spinal axis. As to treatment, the principle I consider safest, is, that it should be divided in two classes, sthenic and æsthenic. Next, consider the diathesis of your patient, and treat accordingly. If complication exists, it will, of course, render the disease more perplexing, and the physician must use his own discretion. I should rely, in uncomplicated cases, upon antiplastics and counter irritants, in the outset; to be followed by tonics and stimulants prudently administered.

